# Vernal keratoconjunctivitis: Current immunological and clinical evidence and the potential role of omalizumab

**DOI:** 10.1016/j.waojou.2023.100788

**Published:** 2023-06-15

**Authors:** Serge Doan, Nikolaos G. Papadopoulos, Jason K. Lee, Salvatore Leonardi, Sara Manti, Susanne Lau, Carmen Rondon, Vibha Sharma, Uwe Pleyer, Xavier Jaumont, Slawomir B. Lazarewicz

**Affiliations:** a Ophthalmology Department of Fondation A de Rothschild and Hôpital Bichat, 25-29 Rue Manin, 75019, Paris, France; b Allergy Department, 2nd Pediatric Clinic, University of Athens, Athens, Greece; c Division of Infection, Immunity and Respiratory Medicine, University of Manchester, Manchester, UK; d Evidence Based Medical Educator Inc., Toronto Allergy and Asthma Clinic, Toronto, Ontario, Canada; e Pediatric Respiratory Unit, AOUP “G. Rodolico-San Marco”, University of Catania, Catania, Italy; f Pediatric Unit, Department of Human Pathology of the Adult and Developmental Age “Gaetano Barresi”, University of Messina, Messina, Italy; g Department of Pediatrics, Division of Respiratory Medicine, Immunology and Critical Care Medicine, Charité Universitätsmedizin Berlin, Berlin, Germany; h Allergy Research Group, Instituto de Investigación Biomedica de Malaga (IBIMA)-Plataforma BIONAND.RICORS “Inflammatory Diseases”, ARADyAL, Malaga, Spain; i Allergy Unit, Hospital Regional Universitario de Malaga, Malaga, Spain; j Lydia Becker Institute of Immunology and Inflammation, University of Manchester, Manchester, UK; k Department of Ophthalmology, CVK, Charité Universitätsmedizin, Berlin, Germany; l Novartis Pharma AG, Basel, Switzerland

**Keywords:** Vernal keratoconjunctivitis, Allergy, IgE, Biologics, Omalizumab

## Abstract

Vernal keratoconjunctivitis (VKC) is a severe ocular allergic disease characterized by chronic inflammation of the cornea and conjunctiva that may lead to loss of visual acuity and blindness. The disease occurs primarily in children and is more common in geographical regions characterized by warm temperatures and high humidity. The clinical manifestations of VKC, when inadequately treated, may lead to severe complications and corneal damage. The prevalence of allergen sensitization, specific serum immunoglobulin E (IgE), and specific tear IgE was reported in approximately 55%–60% of patients with VKC, confirming the involvement of IgE-mediated and non−IgE-mediated mechanisms in the pathophysiology of the condition. This article explores current knowledge on the immunological pathways of VKC and the role of the monoclonal anti-IgE antibody, omalizumab, in its management. The review evaluated the effects of omalizumab beyond the direct IgE-mediated reactions and discusses its potential as a therapeutic target for VKC. Multiple retrospective analyses, case series, and case reports have reported the effectiveness of omalizumab in the management of VKC. A summary of the clinical data from these studies revealed that in children with VKC omalizumab treatment was well tolerated with improvement or resolution of ocular symptoms, reduction in steroid use, and enhancement of quality of life. Omalizumab may serve as a promising treatment option for VKC due to its ability to target both IgE-mediated and non−IgE-mediated pathophysiological pathways. Larger, controlled clinical trials are needed to support these findings.

## Background

Vernal keratoconjunctivitis (VKC) is a severe ocular allergic disease that occurs primarily in children and is characterized by bilateral, recurrent, and chronic inflammation of the cornea and conjunctiva that may lead to loss of visual acuity and blindness.^1^, ^2^, ^3^ The incidence and prevalence rates of VKC vary with geography and season. In colder regions of Europe and the United States, VKC is a rare disease,^4^^,^^5^ with prevalence rates ranging between 0.1% and 0.5%; in hot and humid tropical and sub-tropical regions of Africa, Asia, the Middle East, and Latin America, prevalence rates are higher. In Africa, a wide prevalence of VKC ranging between 2% and 37% has been reported.^5^^,^^6^ VKC usually follows a seasonal pattern with onset in spring, exacerbation in summer, and remission during the autumn-winter period.^7^^,^^8^ Although VKC occurs more frequently in young boys within the first decade of life, it impacts both genders equally in tropical climates.^9^^,^^10^ The condition often resolves spontaneously after puberty but, in approximately 12% of patients, it persists beyond 20 years of age.^7^^,^^11^ Approximately 50% of patients with VKC have an atopic background as assessed by a skin prick test or serum-specific immunoglobulin E (IgE) measurements.^12^^,^^13^

**Fig. 1:**
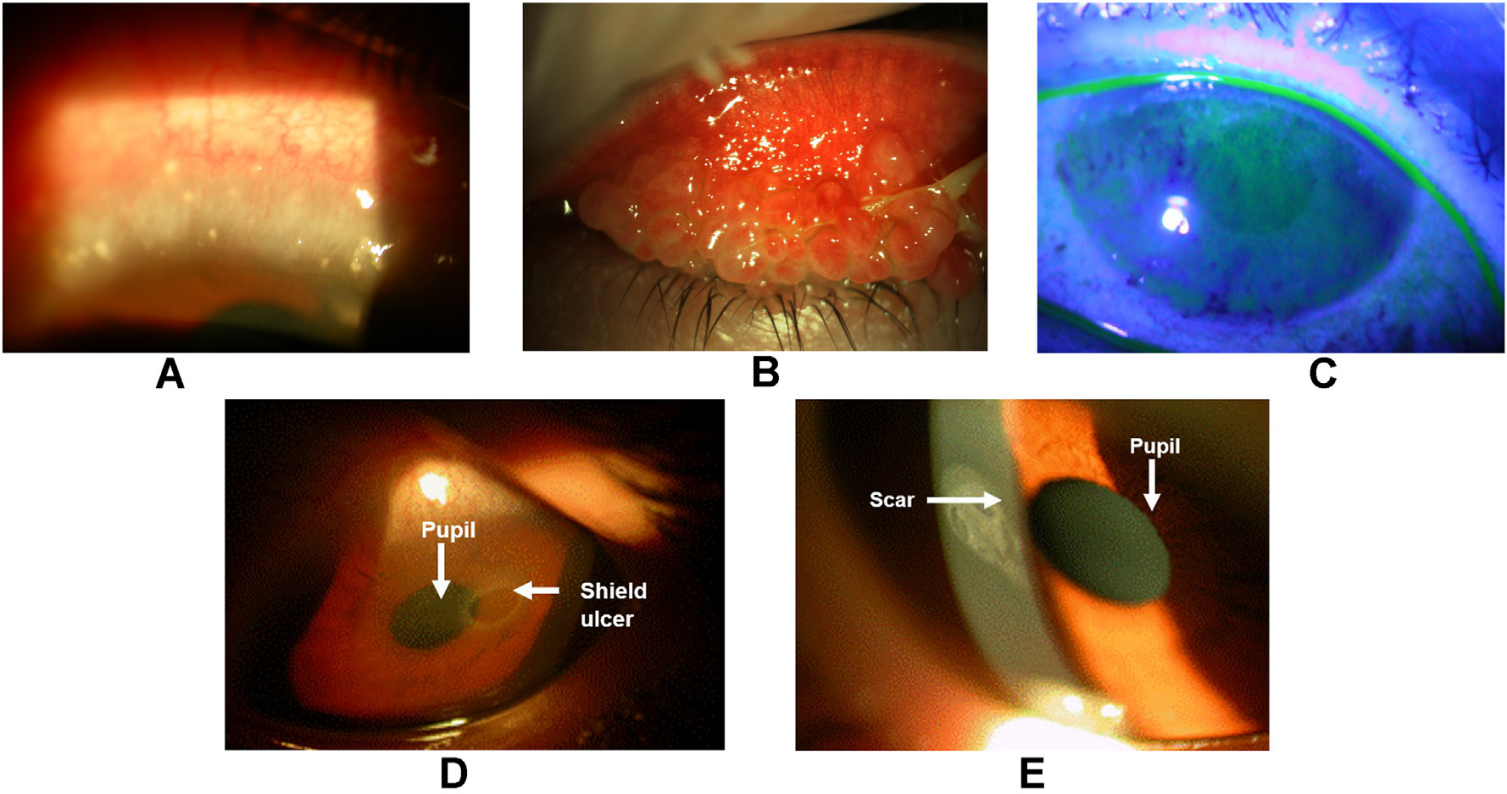
Distinct clinical phenotypes and complications of VKC, A: Limbal swelling with Trantas dots, B: Giant papillae at the upper tarsal conjunctiva, C: Dense superficial punctate keratitis, D: Corneal shield ulcer, E: Corneal scar after a shield ulcer VKC, vernal keratoconjunctivitis.

VKC usually presents as one of 3 clinical phenotypes based on anatomical location. Limbal VKC is the most frequent form (54%) and more common in warmer climates; it presents with limbal swelling and the characteristic Trantas dots at the pericorneal limbus (Fig. 1A). Tarsal VKC is more common in temperate regions and presents with the disease's hallmark giant papillae at the upper tarsal lids (Fig. 1B). The third form is a combination of both.^2^^,^^10^^,^^12^^,^^14^

Patients with VKC often present with symptoms of intense itching, excessive tear production, foreign body sensation, sticky eyes, and conjunctival hyperemia. Intense photophobia and ocular pain have also been reported, which can impact quality of life (QoL).^12^ While most symptoms of VKC overlap with atopic conjunctivitis (AKC), the latter affects both children and adults, is commonly associated with severe atopic dermatitis (AD) of the face and symptoms are usually perennial.^15^^,^^16^

Clinical findings of VKC include conjunctival giant papillae (Fig. 1B), thickened mucus discharge, bulbar conjunctival hyperemia, perilimbal conjunctival hyperpigmentation, and corneal involvement with punctate epithelial erosions in mild cases to vernal shield ulcers or plaques in severe VKC (Fig. 1C–E).^9^^,^^14^ VKC can lead to severe complications in some patients if not treated adequately,^12^ and around 6% of patients develop corneal damage (ulcers, scars, or neovascularization) leading to loss of vision.^9^^,^^12^^,^^17^

Complications of VKC include keratoconus, shield ulcers, pseudogerontoxon, corneal scars and neovascularization, and microbial keratitis. In some patients, prolonged use of topical steroids may lead to iatrogenic complications, including cataracts, ocular hypertension, glaucoma, and corneal infection (Table 1).^10^, ^11^, ^12^

**Table 1:** Symptoms, clinical findings, and complications of VKC.

Symptoms of VKC^10^
Itching (always present)
Photophobia
Lacrimation
Foreign body sensation
Burning
Pain (generally reported with corneal involvement)
White mucus discharge at waking

Clinical findings of VKC	Description
Conjunctival hyperemia^10^^,^^17^	• Bulbar and tarsal conjunctival hyperemia are reported in 86%–90% of patients with VKC • Associated with several etiologies (other than VKC) and hence accurate diagnosis and grading are essential for optimal management of the condition
Conjunctival giant papillae^10^	• Reported in 90% of cases with VKC • Affects the superior tarsal conjunctiva due to prolonged inflammation • Characterized by raised, hyperemic elements • Giant papillae are visible macroscopically • Cobblestone appearance, if found in large numbers • May evolve toward conjunctival scarring
Limbal edema with Trantas dots^10^	• Characterized by the aggregation of epithelial and eosinophil cells at the pericorneal limbus • Typical gelatinous nodular appearance • May evolve toward limbal fibrosis with cysts
Punctate keratitis^10^^,^^18^	• Affects 45% of patients with VKC • Caused mainly by inflammatory mediators from upper tarsal conjunctival giant papillae • Characterized by multiple, subtle, and punctuated small epithelial defects
Perilimbal conjunctival hyperpigmentation^19^	• Multiple, fine, granular, discrete, and dot-like light brown to dark brown pigments • One of the early signs helpful in the diagnosis of VKC; occurs mainly in patients with pigmented skin • Caused by concurrent melanocytic activity and inflammatory reaction of adjacent connective tissues • Immune cells secrete pro-inflammatory cytokines causing melanogenesis and resulting in perilimbal pigmentation

Complications of VKC	Description
Shield ulcer^10^	• Affects 3%–20% of children with VKC • Superficial epithelial ulcer with oval shape, occurring mainly in the superior cornea • May evolve toward plaque formation that will slow the healing process • May lead to opaque corneal scars, severe astigmatism, corneal perforation (exceptional), and amblyopia
Corneal neovascularization^10^	• Refers to the growth of blood and lymphatic vessels in the cornea, which is otherwise avascular • May lead to corneal scarring and a decrease in vision, requiring corneal transplantation in severe cases
Corneal scarring^20^	• Mainly caused by a shield ulcer but can also be related to limbal stem cell deficiency • Can cause vision loss/blurred vision
Pseudogerontoxon^10^	• Commonly found in recurrent limbal disease • Caused by impaired permeability of the limbus and prolonged hyperemia due to the development of Trantas dots, resulting in localized lipid deposits • Resembles gerontoxon, a common finding in geriatric patients • No pathological implications
Keratoconus^10^	• Affects 2%–27% of children with VKC • Mainly related to ocular itching • Irregular conical-shaped cornea due to thinning of the stroma and protrusion of the cornea • May lead to severe astigmatism and impairment of vision
Microbial keratitis^10^	• Rare in patients with VKC, it occurs mainly in those with recurrent shield ulcers and an environment of poor hygiene • Main causes of infection are steroid use, continued epithelial insult, altered immune status, and a reduced level of lactoferrin (bacteriostatic cause)
Cataracts^10^	• Caused by chronic use of topical steroids • Occurs earlier in children and with lower doses of steroids than in adults • Commonly affects the posterior cortex of the lens
Ocular hypertension and glaucoma^10^	• Caused by chronic use of topical steroids • Incidence varies with the frequency and duration of steroid treatment, and the type and formulation of steroids • Children are more prone to increased intraocular pressure than adults • Can lead to blindness

VKC, vernal keratoconjunctivitis

Although the clinical findings and complications of VKC are well characterized, there is a lack of standardized criteria for diagnosis and no uniform definition to grade disease severity. Several methods of grading disease severity and corneal involvement are found in the literature, and each clinician selects a method based on specificity, ease of use, and suitability for their clinical practice.^7^

This state-of-the-art review on the immunological mechanisms involved in the pathophysiology of VKC highlights the IgE-mediated and non−IgE-mediated pathways and the available treatment options, focusing on the ability of the anti-IgE monoclonal antibody, omalizumab, to target IgE and beyond.

## Immunological mechanisms of VKC

The mechanisms governing the pathogenesis of VKC are still under investigation^1^ and are hypothesized to be an interplay of immunological, hormonal, environmental, and genetic factors.^10^^,^^13^ This section will focus on the immunological mechanisms involved in the development of VKC.

VKC is predominantly a type 2 helper T (Th2) cell-mediated disease^9^ and is characterized by both IgE-mediated and non–IgE-mediated immediate and delayed hypersensitivity reactions.^1^^,^^9^^,^^14^

### IgE-mediated reactions

**Fig. 2:**
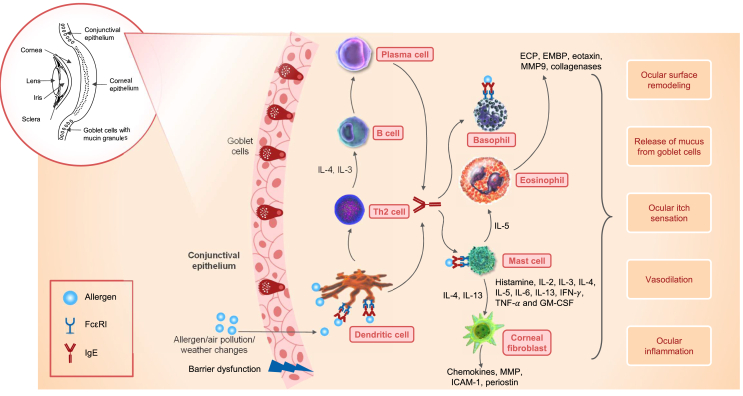
IgE-mediated immunological mechanisms in VKC, ECP, eosinophil cationic protein; EMBP, eosinophil major basic protein; GM-CSF, granulocyte-macrophage colony-stimulating factor; ICAM-1, intercellular adhesion molecule-1; IFN-γ, interferon gamma; IgE, immunoglobulin E; IL, interleukin; MMP, matrix metalloproteinase; Th2, type 2 helper T cell; TNF-α, tumor necrosis factor-alpha; VKC, vernal keratoconjunctivitis

Environmental airborne allergens, such as dust mites or pollen, stimulate the production of IgE, mainly via plasma cells. The allergen-specific IgE binds to high-affinity IgE receptors (FcεRI) on the surface of mast cells, basophils, and dendritic cells, causing degranulation and the release of pro-inflammatory mediators, which are responsible for the symptoms of VKC.^13^^,^^21^ Elevated histamine concentrations, further amplified by reduced histamine degradation by histaminases,^22^ plasma cells, mast cells, macrophages, eosinophils, basophils, fibroblasts, Th2 cytokines (interleukin [IL]-2, IL-3, IL-4, IL-5, IL-8, IL-13, interferon gamma [IFN-γ], and tumor necrosis factor-alpha [TNF-α]), and granulocyte-macrophage colony-stimulating factor (GM-CSF) have been reported in patients with VKC. These cells and mediators play a major role in intensifying ocular inflammation, vasodilation, and the ocular itch sensation, and in the release of mucus from goblet cells (Fig. 2).^13^^,^^14^^,^^23^, ^24^, ^25^, ^26^, ^27^

Additionally, cytokines such as IL-4 and IL-13 activate corneal fibroblasts to secrete chemokines, matrix metalloproteinases (MMPs), and intercellular adhesion molecule-1 (ICAM-1), which results in tissue damage in VKC.^14^ Activated fibroblasts secrete periostin, a downstream protein of IL-13, and are implicated in the pathogenesis of allergic ocular diseases, including VKC.^28^ Formation of conjunctival papillae is attributed to fibroblast production and activation, while nodules in the limbal region are related to the influx of inflammatory cells.^9^^,^^14^

Eosinophils release mediators that contribute to type 2 immune responses in VKC. Eosinophil cationic protein (ECP) and eosinophil major basic protein (EMBP) can disrupt the ocular epithelial barrier. Activated eosinophils secrete chemokines (eotaxin), MMP9, and collagenases that degrade the extracellular matrix, resulting in ocular surface remodeling in VKC.^13^^,^^14^

Although IgE is considered to be a key mediator in ocular inflammation, IgE sensitization (or atopy), assessed by a skin prick test or serum-specific IgE measurements, is reported only in approximately half of the patients with VKC.^12^^,^^13^ Several studies have shown that despite negative systemic-specific IgE levels, high levels of specific IgE may be detected in the tears of patients with VKC, indicating local production of antibodies, known as entopy, which leads to conjunctival sensitization.^13^ This was confirmed in a recent systematic review with meta-analyses based on 33 eligible studies in 2122 patients with VKC.^29^ The meta-analyses showed that the pooled prevalence of allergen sensitization in patients with VKC was 57.7% (95% CI: 52.5%–62.8%). Subgroup analyses of pooled estimates on sensitization reported prevalence estimates of 51.4% for local conjunctival reactivity (based on a conjunctival provocation test), 68.7% for total tear IgE, 58.9% for specific tear IgE, and 58.2% for serum-specific IgE.^29^ Furthermore, high levels of IgE in tears have been reported in patients with VKC in comparison to the 3 other major ocular allergies (AKC, seasonal allergic conjunctivitis, and perennial allergic conjunctivitis).^30^^,^^31^

### Non−IgE-mediated reactions

Several types of non−IgE-mediated reactions occur in VKC, including direct activation of T cells, dendritic cells, enzymes, and MMPs, and non-specific hyper-reactivity.^32^^,^^33^ A wide range of ILs and tumor necrosis factor-alpha and beta 1 (TNF-α and TNF-ꞵ1) explain the expression of a type 1 helper T (Th1)-cell immune response that is linked to chronic and severe forms of VKC.^1^^,^^12^^,^^34^ Thymic stromal lymphopoietin (TSLP) expression is upregulated by allergen-activated conjunctival epithelial cells, which then activate the dendritic cells to promote Th2-mediated allergic responses in VKC.^13^ Increased expression of toll-like receptors (TLRs) were reported that, upon activation, determine the differentiation of Th1 or Th2 response.^35^ The thymus- and activation-regulated chemokine (TARC), also known as CCL17, induces a Th2-dominated inflammatory reaction in patients with VKC.^36^ As part of innate immunity, conjunctival epithelial cells secrete cytokines, chemokines, and adhesion molecules that, upon activation, trigger the influx of Th2 cells and eosinophils to the inflammatory site.^13^ Cytokines and chemokines – such as IL-8, monocyte chemoattractant protein-1 (MCP-1), eotaxin, and RANTES – are produced by mast cells, macrophages, epithelial cells, and fibroblasts that play a role in the non−IgE-mediated pathways in VKC.^33^ Recent evidence suggests a plausible role of natural killer (NK) cells in the pathophysiology and severity of VKC.^13^

It has also been proposed that VKC may not be an isolated ocular disease but rather a systemic inflammatory disease based on the influx of inflammatory proteins and mediators (high-mobility group box-1 [HMGB1] and soluble receptor for advanced glycation end products [sRAGE]) from outside the eye. The concentration of these inflammatory mediators in the serum of children with VKC correlates with the levels in the lacrimal fluid and is also associated with the severity of the disease.^7^

## Treatment of VKC

The main goals of VKC treatment are to interrupt and/or prevent the pro-inflammatory immune response and to stop remodeling to prevent long-term corneal damage and vision loss.^1^^,^^2^ Factors influencing the choice of treatment include disease stage, clinical severity, and duration.^13^

Currently, there are no well-established, standardized treatment algorithms or guidelines for VKC, but some pharmacological options are available that can be individualized based on disease severity.^10^ A systematic review by Leonardi et al (2019) provided a comprehensive overview of the currently available treatments for ocular allergy, including VKC, and suggested treatment recommendations based on the best available evidence in the published literature.^18^

VKC is a persistent and severe allergic corneal disease that might require initial treatment by ophthalmologists in an emergency with subsequent follow-up monitoring and maintenance treatment on a regular basis. Corneal disease experts generally agree that aggressive intervention early in the course of the disease is instrumental in avoiding complications and vision loss.^37^

A multidisciplinary team approach is often recommended, involving an ophthalmologist or a corneal specialist; an allergist to manage specific allergen immunotherapy, if applicable; a pediatrician, considering the age group commonly impacted; and a pneumologist, dermatologist, internist, or an ear, nose, and throat (ENT) specialist to manage any concomitant dermatitis and asthma, rhinitis and any systemic immunosuppressive drugs and their associated complications.^24^^,^^37^ This is similar to other comorbidities and treatment with biologics, which are generally managed by a team of allergists, pneumologists, ENT specialists, or dermatologists based on the type of the comorbid disease and the severity of the condition.^37^ However, given the high prevalence of VKC in developing countries, where access to specialists is limited, patients are often treated only by general practitioners, which may not be optimal.

### Standard therapies

Symptomatic treatment includes avoidance of exposure to bright light, especially sunlight, using sunglasses and a cap; frequent ocular rinsing with saline; and cold compress applications.

Common pharmacological therapies for VKC include anti-allergic and anti-inflammatory treatments such as topical antihistamines, mast-cell stabilizers, non-steroidal anti-inflammatory drugs (NSAIDs), and topical or systemic corticosteroids, depending on disease severity. These medications largely represent palliative therapy rather than targeting the complex immune mechanisms underlying the inflammation. Although relief of symptoms is achieved with steroids, their long-term use is associated with severe ocular and systemic complications, such as increased intraocular pressure, with a tendency toward evolution to glaucoma; cataract formation; and incidence of bacterial, fungal, and viral infections.^18^ While some topical steroids such as loteprednol etabonate and hydrocortisone sodium phosphate may have an improved safety profile, further evaluation is required to support their effectiveness in severe forms of VKC, especially during long-term treatment.^10^

Alternative therapies include topical immunosuppressive drugs, such as cyclosporin A and tacrolimus. These agents are effective in the treatment of VKC symptoms but their long-term use may be associated with tolerability issues as they are frequently reported to have a “burning sensation” and could lead to patients being more prone to opportunistic infections such as papillomavirus, herpes, and molluscum contagiosum.^18^ Hence, VKC treatment remains a challenge for ophthalmologists and allergists.^10^^,^^13^

#### Omalizumab

Novel therapies targeting immunological pathways have been used off-label for VKC, including those targeting IgE. Omalizumab is a humanized monoclonal antibody that binds to free serum IgE and inhibits the binding of IgE to FcεRI on the surface of mast cells, basophils, and dendritic cells. This inhibits the synthesis and release of inflammatory mediators and reduces the presentation of antigens by dendritic cells. In addition, omalizumab inhibits the expression of FcεRI on mast cells and basophils, thereby limiting their reactivity to allergens.^1^^,^^38^ Furthermore, the decrease in the expression of FcεRI on dendritic cells regulates the T-cell responses by reducing Th2 polarization and the associated inflammation.^39^^,^^40^ Lastly, omalizumab decreases the levels of germline CεmRNA on B cells and the surface expression of IL-4R, which result in a reduced allergic response and production of IgE.^40^ This combined effect leads to the attenuation of many markers of inflammation, including eosinophils and levels of GM-CSF, IL-2, IL-4, IL-5, and IL-13. By blocking the binding of IgE to its receptors and decreasing the expression of FcεRI on dendritic cells, omalizumab may also reduce allergen presentation to T cells and the ensuing production of type 2 cytokines in both adaptive and innate immunity.^41^

The effectiveness and tolerability of omalizumab are well established for approved indications such as moderate to severe allergic asthma, nasal polyposis, and chronic spontaneous urticaria (and allergic rhinitis in Japan and Russia), resulting in >1.86 million patient-years of estimated exposure.^38^^,^^42^^,^^43^

VKC is an allergic disease, and omalizumab has proven efficacy in targeting the allergic type 2 inflammatory pathways.^14^ The IgE-mediated pathway in VKC allows omalizumab to be a potential treatment option in the management of all forms of VKC, irrespective of severity.^14^ However, it is currently used mainly in severe forms that do not respond to topical cyclosporin or in patients who need high doses of topical steroids. Severe forms of VKC usually involve long-term treatment spanning several months, so a therapy such as omalizumab with a well-established long-term safety profile would be suitable. Furthermore, VKC mainly affects children and adolescents, populations in which omalizumab is indicated for severe allergic asthma.

**Fig. 3:**
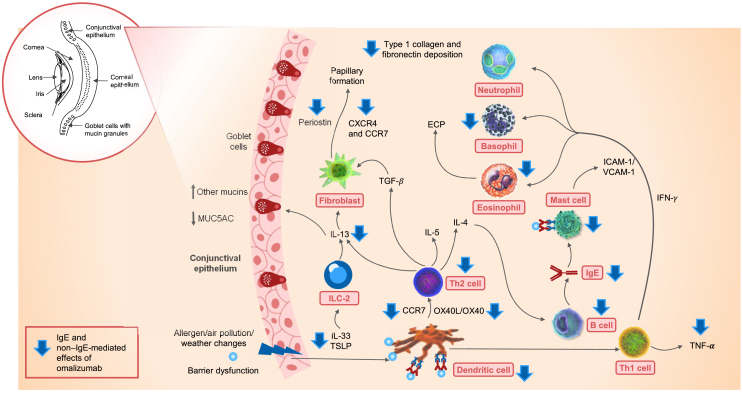
Immunological pathways involved and the downstream effects of omalizumab on allergic inflammation in VKC, CCR7, CC motif chemokine receptor 7; CXCR4, CXC motif chemokine receptor 4; ECP, eosinophil cationic protein; ICAM-1, intercellular adhesion molecule-1; IFN-γ, interferon gamma; IgE, immunoglobulin E; IL, interleukin; ILC-2, type 2 innate lymphoid cell; MUC, mucin; OX40L, OX40 ligand, synonym of CD252 (CD, cluster of differentiation); TGF, transforming growth factor; Th1 cell, type 1 helper T cell; Th2 cell, type 2 helper T cell; TNF-α, tumor necrosis factor-alpha; TSLP, thymic stromal lymphopoietin; VCAM, vascular cell adhesion molecule; VKC, vernal keratoconjunctivitis

Pathophysiological mechanisms of inflammation in VKC that are not directly mediated by IgE also appear to be targeted by omalizumab, as has been reported in other atopic diseases (Fig. 3).^32^^,^^34^ For example, omalizumab treatment for 16 weeks significantly reduced TNF-α levels compared with a placebo in patients with allergic rhinitis.^44^ In patients with allergic asthma, omalizumab therapy for 12–16 weeks increased eosinophilic apoptosis, reduced ECP levels, and decreased the number of T cells producing GM-CSF, IL-2, and IL-13 in peripheral blood.^45^, ^46^, ^47^ Similarly, in patients with allergic asthma, omalizumab therapy for 16 weeks decreased eosinophils in the peripheral blood and tissues and decreased the number of clusters of differentiation (CD3^+^, CD4^+^, and CD8^+^) cells, B cells, and IL-4-positive cells.^46^ *In vitro*, omalizumab decreased the deposition of type 1 collagen and fibronectin, which are implicated in the pathophysiology of VKC.^46^

In patients with severe asthma, serum MMP9 levels decreased significantly at 12, 24, and 36 months after initiating omalizumab therapy.^48^ In comparison to the placebo, omalizumab decreased the levels of TSLP, OX40L (also known as CD134), TARC (or CCL17), and IL-9, while the tolerogenic cytokine, IL-10, increased in patients with severe AD.^39^ In patients with severe allergic asthma, omalizumab decreased serum IL-33 and IL-13 levels; this led to the inhibition of fibrocyte recruitment and proliferation, and myofibroblast transformation through the IL-33/ST2 axis. Furthermore, omalizumab downregulated the CXC motif chemokine receptor 4 (CXCR4) and CC motif chemokine receptor 7 (CCR7) expression of fibrocytes, which could suppress fibrocyte recruitment within the lungs.^49^

The anti-IgE treatment restored the capacity of plasmacytoid dendritic cells (pDCs), impaired by cross-linking of IgE–FcεRI, to induce regulatory T cells (Tregs) in vitro in atopic patients.^50^ In children with allergic asthma and rhinovirus illness, omalizumab reduced the surface expression of FcεRI on pDCs (and on mast cells). By blocking IgE binding to the receptor, omalizumab also prevents receptor cross-linking by the allergen. These effects of this anti-IgE therapy may potentially enhance IFN-α responses, thereby limiting the cell-to-cell spread of respiratory viruses and subsequently diminishing the severity of the infection and associated level of illness.^51^ Finally, omalizumab significantly reduced serum periostin levels at 4 and 8 weeks of treatment in patients with asthma, thereby lowering the inflammatory responses.^28^

Biologics other than omalizumab (e.g., anti−IL-4 and anti−IL-5 monoclonal antibodies) that target immune mechanisms have not been studied extensively in VKC.^52^ Dupilumab (an anti−IL-4 monoclonal antibody) has been evaluated in patients with atopic keratoconjunctivitis but not VKC. While this biological agent has shown potential benefits in treating severe forms of ocular allergy, there have been several reports of dupilumab worsening ocular surface disease and increasing the risk of blepharoconjunctivitis in patients with AD.^14^^,^^53^

### Clinical findings of omalizumab in VKC

A comprehensive literature search was performed using PubMed and EMBASE databases for articles that reported the use of omalizumab in VKC, published from inception to June 27, 2022 (updated on November 30, 2022). The medical subject headings and search terms used were: “omalizumab”, “Xolair”, “anti-IgE”, “vernal keratoconjunctivitis”, “VKC”, and “allergic conjunctival diseases”. The studies were filtered by clinical trial, publication type, and language (English). Any other potential studies were identified by manually searching based on the eligibility criteria. The search found a total of 18 articles on the use of omalizumab in 46 patients with VKC, and this included 4 retrospective analyses, 2 case series, and 12 case reports (Table 2).

**Table 2:** Review of the literature: Clinical studies in VKC.

Study reference (first author, year)	No. of patients	Age in years/sex (M/F)	Atopic comorbidity	Omalizumab dose	Treatment duration	Outcome/control of VKC
Retrospective analyses						
Rossberg, 2020^2^	3	10/M	Allergic rhinoconjunctivitis and asthma	300 mg/2 weeks	11 months	Total control in all 3 patients
		6/M	Allergic rhinoconjunctivitis	Maximum of 300 mg/4 weeks	7 months (across 3 years)	
		7/M	Allergic rhinoconjunctivitis, AD, and asthma	300 mg/2 weeks	6 months	
Doan, 2019^57^	5	6−13/M	All had asthma and rhinitis; 4 had eczema	450−600 mg/2 weeks	>12 months	Total control: 1 patient Partial control: 3 patients No control: 1 patient
Coutu, 2019^57^	10	7−19/9 M and 1 F	9 patients sensitized to airborne allergens and 4 to food allergens	Based on weight and total serum IgE levels	Not available (mean ± SD follow-up after first injection: 62.9 ± 33.85 weeks)	This was a QoL study. Total control (no corneal ulcers during the treatment period) with good QoL improvements in all 10 patients
Doan, 2016^32^	4	7−13/M	All had asthma and 1 had severe eyelid eczema	450–600 mg/2 weeks	Median: 33 months in 3 patients	Total control: 3 patients No control: 1 patient (treatment discontinued)
Case series						
Silva, 2019^60^	3	6/M 23/F 15/M	All patients had severe asthma and allergic rhinitis	Regular dosing for asthma	180 days	Total control in all 3 patients
Occasi, 2017^59^	4	6/M	Eczema in 1 patient and persistent rhinitis in 2 patients	225 mg/2 weeks	6 months	Total control in all 4 patients
		8/M		300 mg/4 weeks		
		11/F		225 mg/4 weeks		
		9/M		225 mg/4 weeks		
Case reports						
Zengarini, 2022^61^	1	13/M	Severe AD	300 mg/month	3 months (ongoing)	Total control
Manti, 2021^1^	2	12/M	Severe allergic asthma	450 mg/2 weeks	9 months	Total control in both patients
		10/M		600 mg/2 weeks		
Tardino, 2020^62^	2	13/NA	Asthma	Based on weight; every 2 weeks	16 weeks	Total control in both patients
		10/NA				
Limao, 2020^63^	2	17/M	Eczema and allergic rhinitis	Dosing details NA	5 years	Total control in both patients
		10/M	Eczema, allergic rhinitis, and asthmaweeks		10 months (ongoing)	
Simpson, 2019^64^	1	54/M	Seasonal allergic rhinoconjunctivitisweeks	300 mg	Single dose	Total control
Santamaria, 2018^65^	1	15/F	Rhinitis	225 mg/2 weeks, reduced to 150 mg/2 weeks after 24 monthsweeks	54 months	Total control, with relapse upon discontinuation
Picardi, 2016^66^ Heffler, 2016^54^	2	9/F	None	300 mg/month	6 months	Total control
		21/M	AD	600 mg/month		Partial control
Occasi, 2015^67^	1	15/M	Asthma and AD	600 mg/2 weeks	3 months	Total control
de Klerk, 2013^56^	1	12/M	Allergic asthma, severe rhinoconjunctivitis, and eczema	300 mg/month	18 months	Total control
Sanchez, 2012^3^	1	15/M	Asthma, rhinitis, and AD	300 mg/2 weeks	9 months	Total control
Williams, 2005^37^	3	33/M	Asthma, rhinitis, and AD	Based on weight and total serum IgE levels	Not specified	Total control
		67/F				No changes
		59/F				Total control

AD, atopic dermatitis; F, female; IgE, immunoglobulin E; M, male; NA, not available; QoL, quality of life; SD, standard deviation; VKC, vernal keratoconjunctivitis

The clinical data from the results of the literature search showed that, in patients with severe VKC unresponsive to standard therapies, omalizumab treatment was well tolerated and improved or resolved ocular symptoms; this was followed by complete or partial remission, gradual topical steroid dose reduction and termination, improvement of any atopic comorbidities in most cases, and enhancement of QoL in children (Table 2). It is noteworthy that there were very few treatment failures and the reasons for those failures may be attributed to the absence of atopy/allergic sensitization or low total serum IgE levels.^32^ However, these cannot be considered as defining criteria for initiating omalizumab as there were several case reports where patients without atopy or with low serum IgE levels were successfully treated with omalizumab.^54^ The omalizumab dose in these studies ranged from 225 mg/2 weeks to 600 mg/2 weeks, and the treatment duration was between 3 and 60 months. The age range of patients included in the studies was 6–67 years, with the majority of patients being children or adolescents aged 6–15 years; 3 patients were over 50 years old.

VKC is associated with several atopic comorbid conditions, including asthma, rhinitis, allergic rhinoconjunctivitis, AD, eczema, and food allergy. In patients with VKC, around 85% of patients had allergic comorbidity.^55^ Rhinitis was the most frequent comorbidity, followed by asthma and allergic dermatitis.^55^^,^^56^ While omalizumab is not indicated for the treatment of VKC, prescribing it for the treatment of allergic comorbidity has allowed its effectiveness in VKC to be demonstrated. In patients with VKC without any atopic comorbidities, treatment with omalizumab is not possible in some countries, creating an unmet clinical need.

In most of the studies reviewed, the outcomes of the use of omalizumab in patients with VKC were evaluated using several methods; these included the ocular visual analog scale (VAS),^21^^,^^32^^,^^57^ clinical grading (Bonini scale),^2^^,^^58^ resolution of AD and VKC symptoms,^3^^,^^59^ Juniper's rhinoconjunctivitis QoL score,^21^^,^^56^ and Asthma Control Test (ACT) scores^32^ in patients with asthma. Treatment responses were graded based on ophthalmologic examination and changes in ocular symptoms and inflammatory flares.^1^^,^^32^ “Total control” refers to the absence of symptoms and inflammatory flares, “partial control” indicates a decrease in the intensity of symptoms and/or the number of flares, and “no control” denotes that there was no improvement in disease status (Table 2).^32^

## Future research

Multiple small studies have established the important role of IgE in VKC and the beneficial effect of omalizumab in VKC, prompting larger studies and further exploration of the underlying disease mechanisms in VKC and the role of anti-IgE therapy, mainly in patients without high systemic IgE. Since locally high levels of IgE have been reported in tears, detection of tear IgE levels may help guide treatment decisions in patients with a negative skin prick test and low serum-specific IgE levels. The absence of allergic sensitization as a risk factor for treatment failure with omalizumab also warrants further research.

Large, prospective, randomized, and controlled trials to confirm the efficacy of omalizumab in VKC patients are warranted, especially to help determine the optimal dose and duration to achieve maximal benefit. Other research areas in VKC where there are data gaps with omalizumab include long-term effectiveness and safety in children <6 years old, health-economic analyses, and evaluations of possible relapse after discontinuation.

## Conclusions

Given the unmet therapeutic need to manage moderate and severe VKC in addition to the complications associated with this condition, omalizumab appears to benefit pediatric patients and is a promising treatment option based on its ability to target IgE-mediated and non–IgE-mediated pathways. Larger, controlled clinical trials are needed to support these positive findings from small studies of omalizumab in the treatment of VKC.

## Abbreviations

AD, atopic dermatitis; CCR7, CC motif chemokine receptor 7; CD, cluster of differentiation; CXCR4, CXC motif chemokine receptor 4; ECP, eosinophil cationic protein; EMBP, eosinophil major basic protein; ENT, ear, nose, and throat; GM-CSF, granulocyte-macrophage colony-stimulating factor; HMGB1, high-mobility group box-1; ICAM-1, intercellular adhesion molecule-1; IFN-γ, interferon gamma; IgE, immunoglobulin E; IL, interleukin; MCP-1, monocyte chemoattractant protein-1; MMP, matrix metalloproteinase; NSAID, non-steroidal anti-inflammatory drug; OX40, synonym of CD134 (CD, cluster of differentiation); pDC, plasmacytoid dendritic cell; QoL, quality of life; sRAGE, soluble receptor for advanced glycation end products; TARC, thymus- and activation-regulated chemokine; Th1 cell, type 1 helper T cell; Th2 cell, type 2 helper T cell; TLR, toll-like receptor; TNF-α, tumor necrosis factor-alpha; TNF-ꞵ, tumor necrosis factor-beta; Tregs, regulatory T cells; TSLP, thymic stromal lymphopoietin; US, United States; VAS, visual analog scale;VKC, vernal keratoconjunctivitis.

## Acknowledgments

The authors thank Preethi B and Ian Wright (CONEXTS-Medical & Knowledge Solutions, India and UK) for providing medical writing support in accordance with the Good Publication Practice 2022 (GPP2022) guidelines (https://www.ismpp.org/gpp-2022). The study and the writing support was funded by Novartis Pharma AG, Basel, Switzerland).

## Availability of data and materials

Not applicable as this is a review article and not an original research.

## Author contributions

All authors have contributed equally to the design, review, and preparation of the manuscript.

## Ethics statement

Not applicable, as this is a review article and not an original research.

## Authors’ consent for publication

I confirm that all authors listed on the title page have agreed to the submission and consequent publication of the manuscript, post-review by the editorial board.

## Declaration of competing interest

**Serge Doan** reports the receipt of consulting fees from 10.13039/100007816Alcon, Bausch & Lomb, Horus, 10.13039/100004339Sanofi, 10.13039/501100004286Santen, and Thea.

**Nikolaos G. Papadopoulos** reports the receipt of grants from Capricare, Nestle, Numil, and Vianex; and consulting fees from Abbott, 10.13039/100006483AbbVie, 10.13039/100004325AstraZeneca, 10.13039/100004330GlaxoSmithKline, HAL, Medscape, Menarini/Faes Farma, 10.13039/100016259Mylan, 10.13039/100004336Novartis, Nutricia, OM Pharma, and 10.13039/100009857Regeneron/10.13039/100004339Sanofi.

**Jason K. Lee** reports the receipt of grants from 10.13039/100004339Sanofi, 10.13039/100009857Regeneron, 10.13039/100006483AbbVie, Astra Zeneca, and 10.13039/100004330GlaxoSmithKline. He has received consulting fees, speaker payments, and travel and meeting support from Astra Zeneca, 10.13039/100009857Regeneron, 10.13039/100004330GlaxoSmithKline, Pfizer, 10.13039/501100014565Bausch Health, Medexus, 10.13039/100004339Sanofi, 10.13039/100006483AbbVie, 10.13039/100004336Novartis, ALK, Valeo, Miravo, and 10.13039/100004331Johnson and Johnson. He has served on advisory boards for Sanofi, GlaxoSmithKline, Novartis, Regeneron, and AstraZeneca.

**Susanne Lau** reports the receipt of consulting fees and payments for lectures from Sanofi-Aventis and has participated in advisory board meetings for 10.13039/100009946Allergopharma, 10.13039/501100011831DBV, 10.13039/100004339Sanofi, and Leo Pharma.

**Vibha Sharma** reports the receipt of grants for conducting research, payments for expert testimony, honoraria, speaker fees, travel support, and medical writing payments from Aimmune Therapeutics. She has also received payment from Santen for expert testimony. She is a member of the Paediatric Steering Group within the British Society of Allergy and Clinical Immunology, and the European Academy of Allergy and Clinical Immunology, Ocular Allergy Working Group.

**Uwe Pleyer** reports the receipt of grants from the 10.13039/501100000780European Union. He has received honoraria for lectures from AbbVie, Allergan, Alimera, Bayer, Novartis, Pfizer, Roche, Santen, and Thea. He is a board member of Deutsche UVEITIS Arbeitsgemeinschaft DUAG e. V and Head of the board of Uveitis/DOG section.

**Carmen Rondon, Salvatore Leonardi,** and **Sara Manti** have no conflicts of interest to disclose.

**Xavier Jaumont** and **Slawomir B. Lazarewicz** are employees of Novartis.
